# Multi-Objective AI Optimization of Plastic Waste Pyrolysis Integrating Energy Return on Investment for Circular Polymer Recycling

**DOI:** 10.3390/polym18091062

**Published:** 2026-04-28

**Authors:** Abhirup Khanna, Bhawna Yadav Lamba, Sapna Jain, Anushree Sah, Sarishma Dangi, Abhishek Sharma, Jun-Jiat Tiang, Sew Sun Tiang, Wei Hong Lim

**Affiliations:** 1 School of Computer Science, University of Petroleum and Energy Studies, Dehradun 248007, India; akhanna@ddn.upes.ac.in (A.K.); asah@ddn.upes.ac.in (A.S.); 2 Applied Science Cluster (Chemistry), School of Advanced Engineering, University of Petroleum and Energy Studies, Dehradun 248007, India; 3 Department of Computer Science and Engineering, Graphic Era Deemed to Be University, Dehradun 248002, India; sarishma.cse@geu.ac.in (S.D.); abhisheksharma.cse@geu.ac.in (A.S.); 4 Centre for Wireless Technology, CoE for Intelligent Network, Faculty of Artificial Intelligence & Engineering, Multimedia University, Persiaran Multimedia, Cyberjaya 63100, Malaysia; 5 Faculty of Engineering, Technology and Built Environment, UCSI University, Kuala Lumpur 56000, Malaysia; tiangss@ucsiuniversity.edu.my (S.S.T.); limwh@ucsiuniversity.edu.my (W.H.L.)

**Keywords:** plastic waste, pyrolysis, AI optimization, circular economy, energy efficiency, sustainable polymer recycling

## Abstract

A rapid accumulation of plastic waste has created an urgent need for efficient and sustainable recycling technologies. Among various approaches, pyrolysis stands out as promising method of thermochemical recycling of plastic waste; however, the process needs optimization and further research to make it more energy-efficient and sustainable. The conventional approaches for optimization focus on the enhancement of yield, only overlooking efficiency and system-level sustainability. In this study, a machine learning-enabled surrogate-assisted multi-objective artificial intelligence (AI) optimization framework is developed for plastic pyrolysis to maximize product recovery and minimize energy consumption. The model integrates energy return on investment (EROI) and higher heating value (HHV) into process design. A curated dataset of 312 experimental cases covering polyolefins, PET, nylon, and mixed plastics was used to train multiple machine learning algorithms, such as polynomial regression, Gaussian process regression, and Random Forest models. The Random Forest algorithm demonstrated superior predictive robustness across oil yield, HHV, char formation, and EROI. Pareto front analysis using NSGA-II revealed that moderate reaction severities (400–450 °C, 40–70 min) maximize net energy performance while minimizing solid residues. The conditional variational autoencoder as a GenAI model was incorporated to work as a generative proposal engine, which enhances the exploration of chemically feasible operating regions under uncertainty-aware active learning. The integration of techno-economic and life-cycle assessment demonstrates that energy-positive configurations outperform high-yield scenarios, achieving IRR > 15%, energy intensity < 10 MJ kg^−1^, and CO_2_ reductions up to 47% relative to incineration. The proposed framework establishes a data-driven methodology for aligning polymer pyrolysis optimization with circular economy and energy sustainability objectives.

## 1. Introduction

Plastics have become essential materials across a wide range of sectors, including important sectors such as construction, healthcare, electronics, automotive, and packaging [1,2]. Their unmatched diverse properties like light weight, high strength, adaptability, and cost efficiency make them suitable for applications in different sectors, thus facilitating technological progress and economic development. Rapid urbanization and industrialization have led to an exponential increase in global plastic production [3]. Considering the existing growth rate, plastic waste generation is projected to reach 26 billion tons by 2050 [4,5,6].

The conventional plastics, such as polyethylene (PE), polypropylene (PP), polystyrene (PS), polyvinyl chloride (PVC), and polyethylene terephthalate (PET), are mainly derived from petroleum-based polymers [5] derived from fossil fuels, thereby raising concern about long-term sustainability [7]. In addition, the improper waste management system has resulted in substantial buildup in landfills and across natural ecosystems [8].

The lack of competent recycling, waste management and circular economy approach raises serious concerns, such as greenhouse gas emission, resource exhaustion and long-lasting pollution [9].

These existing conditions necessitate the development of sustainable waste management practices and alternatives.

On the other hand, there are some advanced technologies that facilitate the improvement of recycling efficiency and reduce environmental impact. Chemical recycling methods, such as pyrolysis [10,11], gasification [12], and hydrothermal processing [13], support the reclamation of useful material from waste plastics [14]. In addition, the application of metal-based catalysts improves the efficiency of the conversion of waste plastic into useful hydrocarbons [15]. Among the above technologies, pyrolysis has appeared as the most sustainable and efficient method for plastic waste valorization [16,17,18,19,20]. When compared to incineration, pyrolysis causes less harmful emissions as it occurs in the absence of oxygen. In contrast to mechanical recycling, pyrolysis does not require sorting and segregation; rather, it can directly process the multilayered missed waste plastic into useful products, including pyrolysis oil, gas, and char.

The process converts waste plastics into useful hydrocarbon products that can be used as fuel in neat or blended form, thereby reducing dependency on fossils fuel, mitigating environmental pollution, and promoting circular economic principles, thus aligning it as a technologically strong and environmentally advantageous solution for sustainable waste plastic management.

India has established one of the most structured regulatory frameworks for pyrolysis, with clearly defined standard operating procedures (SOPs), state-level classifications, specified minimum plant capacities, and a balanced Extended Producer Responsibility (EPR) mechanism. In comparison, the United States maintains a fragmented regulatory landscape: while some states categorize pyrolysis as a manufacturing activity, federal regulations often continue to classify it as incineration, necessitating stringent permitting requirements. Similarly, the European Union has yet to formally incorporate chemical recycling within its Waste Framework Directive. China’s 2018 ban on plastic waste imports significantly altered global plastic waste flows and reshaped domestic pyrolysis implementation strategies [21,22,23,24,25,26,27,28].

In recent years, artificial intelligence and machine learning have emerged as transformative tools for advancing pyrolysis research by addressing these nonlinearities and accelerating process optimization [29,30,31,32]. AI-driven models can analyze extensive experimental datasets to capture hidden relationships between operational variables and chemical outcomes, thereby allowing predictive mapping of product distributions without exhaustive experimentation. Supervised learning algorithms such as Random Forests, Support Vector Machines, Gradient Boosting, and Neural Networks have been successfully applied to predict oil yield, HHV, and gas composition from heterogeneous polymer mixtures. Similarly, unsupervised and hybrid learning methods facilitate the clustering of feedstock types and anomaly detection. Surrogate modeling and evolutionary algorithms have revolutionized multi-objective optimization in pyrolysis [33,34]. The models enable the identification of Pareto-optimal conditions that balance oil yield, energy efficiency, and char minimization. The integration of surrogate modeling algorithms with sustainability metrics, such as EROI, extends their impact from empirical optimization to holistic process design. The integration of data science with thermochemical engineering shifts the approach from trial-and-error reactive experimentation to intelligent, predictive, and adaptive control. This enables improved optimization of the process, making it more scalable, energy-efficient, and inclined towards circular economy.

Pyrolysis is another promising line of turning heterogeneous plastic waste into relevant economic products, e.g., pyrolysis oil, synthesis gas and char. However, until recently, optimization has been based on empirical experimentation, which is labor-intensive, time-consuming, and, by the nature of any empirical task, pushed towards the constraints of feedstock variability and operating parameter range. Traditional methods tend to focus on raw efficiencies of mass-conversion at the expense of other, more holistic measures of sustainability, such as higher heating value (HHV), energy return on investment (EROI), and the minimization of char. This gap highlights how artificial intelligence (AI) and multi-objective frameworks could deliver and balance the conversion efficiency of masses with energy recuperation strength. By modeling multiple pyrolysis outcomes (oil yield, HHV, and the formation of char), sophisticated machine learning (ML) surrogate models revolutionize the optimization paradigm by enabling multifactor–multitarget optimization: predicting multiple outputs with high precision. These models can provide fast, data-based, no-go forecasting without the use of a large volume of trial-and-error experimentation, thus systematically exploring the multidimensional process space. Such models, when applied together with surrogate-assisted evolutionary algorithms, allow the identification of Pareto-optimal trade-offs, thus determining the conditions under which both the oil yield and energy content is maximized and the undesirable by-products minimized. In contrast to single-objective approaches, this approach integrates sustainability in the process design itself, which not only ensures high performance, but also ensures energy efficiency. Models incorporating EROI and fuel quality (usually with the help of HHV) fundamentally transform this landscape by showing that those conditions that are optimized with regard to yield alone may not provide the best energetic or economical results. The combination of surrogate modeling with active learning through a closed-loop framework enables the discovery process to proceed further and even faster by adapting the choice of regions to explore in the parameter space so that they can be both underexplored and maximally valuable (minimizing the number of experimentations, with improved scalability and robustness). In this work, generative artificial intelligence is used only to create new input settings for the plastic waste pyrolysis optimization problem. It does not predict process results, and it does not rank or choose the best solutions. Instead, it works as a structured sampling tool that proposes possible operating conditions and feedstock combinations. These proposed inputs are then evaluated by a trained Random Forest surrogate model.

## 2. Research Questions

○ RQ1: How accurately can machine learning surrogate models predict oil yield, HHV, char formation, and EROI from heterogeneous polymer feedstocks?
○ RQ2: What operating conditions and feedstock characteristics enable Pareto-optimal trade-offs between liquid yield, energy efficiency, and residue minimization?
○ RQ3: How does incorporating EROI reshape optimal pyrolysis design compared to yield-only optimization?
○ RQ4: To what extent can generative sampling under active learning improve the exploration efficiency of high-performing pyrolysis configurations?

## 3. Methodology

### 3.1. Surrogate Model Development

The study was eventually intended to hasten the optimization of the pyrolysis of plastic waste with the application of surrogate modeling with a focus on three models of predictive process: polynomial regression, Random Forest and Gaussian process regression (Kriging). The work included studies only if they reported explicit operating conditions (temperature, residence time, reactor type), quantified product yields, and the elemental composition of feedstocks. Catalytic or co-feeding systems were excluded. Continuous variables were normalized using min–max scaling. Missing values were excluded if critical; minor gaps were imputed via median substitution within polymer classes. Both the frameworks were used to predict the main process product of oil yield, char yield and oil higher heating value (HHV) based on operation conditions (temperature, residence time) and the composition of the feedstock (carbon, hydrogen, and nitrogen content and input HHV). The choice of model was based on a trade-off between interpretability, predictive performance and the ability to deal with heterogeneous data on pyrolysis. Polynomial regression gave an easily understood benchmark that could cover some nonlinearity in the form of higher-order terms, but accuracy in prediction was limited in areas with multifaceted interactions, particularly when predicting HHV. Random Forest proved the most sustainable and robust model as, regardless of outputs, it turned out to be the most stable model and yielded the best performance compared to the two other models. Its architecture allowed the representation of nonlinearities and interactions simultaneously, with no overfitting, and thus made it very appropriate to systems like pyrolysis, where non-additivity is intrinsic to the system and its interactions were reliable. Meanwhile, the Kriging model did not converge significantly with the dataset but instead collapsed around its predictions, illustrating the weaknesses in its usage with noisy, discontinuous data associated with heterogeneous mixed plastic feedstocks and different experimental conditions. In a complementary fashion to prediction, the surrogate modeling provided mechanistic insights via an analysis of feature importance (via SHAP values). The depletion of oil higher heating value occurred almost under exclusive dominance of the initial carbon content of the feed, and oil yield correlated mainly with temperature and residence time. It was not only a matter of process severity, but also of feedstock mix, that influenced whether char was yielded, and the outcome of a prudent interplay of feedstock chemistry and operating conditions was the energy return on investment (EROI). Neural Network models were not adopted in the study due to the relatively small dataset size (*n* = 312), which limits their generalization capability and increases overfitting risk in high-dimensional nonlinear architectures. Table 1 presents the functional description of all computational models. The work prioritized interpretability to enable feature importance and SHAP-based mechanistic insights, which are less transparent in deep learning models. Furthermore, the heterogeneous and discontinuous nature of mixed-polymer pyrolysis data favors ensemble tree-based methods, which are more robust to sparse, noisy, and non-smooth response surfaces.

### 3.2. Multi-Objective Optimization Framework

A multi-objective optimization framework has been established in this study to help negotiate the trade-offs of the performance measures in the pyrolysis process, namely the oil yield, oil higher heating value (HHV), char yield and energy return on investment (EROI). The traditional single-objective methods in pyrolysis are typically guided by maximizing the yield of liquid products, which is not an efficient factor since it does not consider energy efficiency and less production of products. To overcome this drawback, the framework uses surrogate-aided evolutionary algorithms: a machine learning model is combined with a genetic algorithm to sample a high-dimensional space which is too complex to sample with the method of evolutionary algorithms alone. Surrogate model Random Forests are selected due to their reliability and high computational efficiency and the fact that they can be used as a fast predictor of the results of pyrolysis, theoretically replacing more expensive or time-intensive experiments and significantly accelerating the assessment of candidates and solutions. Interpretability can also be improved through feature importance analysis showing the connections between the process conditions and feedstock composition and performance, directing the optimizer to the significant variables. Its optimization algorithm is the non-dominated sorting genetic algorithm (NSGA-II), which is well adapted in the identification of Pareto fronts in multi-objective contexts. Although the objective set includes the variables oil yield, HHV, char yield and EROI, the framework aims to maximize liquid fuel recovery and energy quality simultaneously with minimizing char production and having a positive net energy output. The method gives a wide sampling of Pareto-optimal solutions that would have a different trade-off of the performance goals. As an example, conditions that make it optimal to maximize yield might not be the same as those to maximize EROI, showing the advantages of a multi-objective approach. Further, the framework also uses a closed-loop feature of active learning: surrogate models may be progressively improved using additional data in those parts of the design space where predictions have the greatest potential, thereby encouraging the iterative optimization of prediction accuracy and the containment of experimental workload.

The input variables in the study comprised the type of plastics (LDPE, Nylon-6, polypropylene, HDPE, polyethylene terephthalate and mixed plastics), ultimate analysis of plastics (percentage of carbon, percentage of hydrogen and percentage of nitrogen), type of reactor (fixed-bed, semi-batch, fluidized-bed, batch), pyrolysis temperature (°C), and time (minutes). A thorough literature review was part of the technique used for this study in order to collect relevant data from a variety of peer-reviewed journal articles. The main goal was to gather information about mentioned input parameters from a variety of theoretical and experimental investigations. Relevant information was found, chosen, and extracted using a methodical process. To guarantee accuracy and consistency, the gathered data was thereafter thoroughly examined, augmented, and curated. The basis for comparative analysis and interpretation was the carefully selected dataset [35,36,37,38,39,40,41,42,43,44,45,46,47,48,49].

### 3.3. Generative AI-Assisted Optimization and Decision-Support Framework

The generative model is trained using a carefully selected dataset of Pareto-optimal and near-optimal solutions obtained from the multi-objective optimization stage. Each training example includes only input variables such as pyrolysis temperature, residence time, reactor type and plastic feedstock category. A conditional variational autoencoder (CVAE) is used to learn the probability distribution of feasible input combinations under desired performance conditions. It samples from its latent space; the model can generate new operating configurations that are statistically like high-performing solutions but not limited to previously tested points. To ensure physical realism, all generated variables are restricted to experimentally and industrially valid ranges for temperature, time, and feedstock composition, thereby preventing the occurrence of unrealistic and infeasible operating conditions. Every candidate configuration produced by the generative model is then evaluated using the Random Forest surrogate model, which quickly predicts pyrolysis outcomes. The implementation of the GenAI model improves exploration efficiency by replacing random or heuristic sampling methods. When compared to uniform random sampling, the proposed approach focuses on chemically meaningful regions of the design space. The use of CVAE increases the chances of finding balanced solutions that meet multiple objectives, such as high yield, good energy efficiency, and low residue formation. Moreover, the GenAI model is used as part of an active learning and adaptive sampling system to make multi-objective optimization of plastic waste pyrolysis more efficient. Traditional surrogate-based optimization methods often start with fixed or random sampling, which can result in wasted evaluations and slow down convergence, especially when the design space is large and complex. The GenAI model focuses on parts of the input space where the surrogate model has high prediction uncertainty; the Pareto front has limited coverage where there is great expected improvement for important objectives (maximizing energy return on investment and minimizing char production). Instead of proposing small variations in already tested solutions, the generative model learns patterns from previously evaluated data and suggests new configurations that are more likely to provide useful and informative results. All generated candidates are evaluated using the Random Forest surrogate model. Their predicted performance and uncertainty are analyzed.

## 4. AI-Optimized Pyrolysis: TEA–LCA Integration

To translate the optimization framework into actionable sustainability insights, the Pareto-optimal outputs of oil yield, HHV, char formation, and EROI were embedded within a combined techno-economic and life-cycle assessment (TEA–LCA) framework. Table 2 presents the physicochemical properties of the plastic feedstocks considered, including elemental composition and input higher heating value, highlighting the chemical heterogeneity of polymers such as polyolefins, PET, nylon, and mixed plastic streams and their distinct pyrolytic behaviors. A comparison of the predictive performance of the surrogate models suggests the better accuracy and robustness of the Random Forest model in predicting oil yield, HHV, char formation, and EROI, justifying its selection for subsequent optimization (Table 3). Table 4 translates Pareto front results into representative operating conditions, thereby illustrating how different trade-off scenarios (high-yield, balanced, and energy-positive) emerge from multi-objective optimization. Table 5 explains the functions and constraints of each computational component. A link of optimization outcomes to circular polymer economy objectives, demonstrating how energy efficiency, waste minimization, and resource recovery are simultaneously addressed within the proposed framework (Table 6). Table 7 demonstrates that energy-positive and balanced Pareto-optimal configurations outperform high-yield operation in both economic and environmental performance. Incorporating EROI into the optimization framework enables scenarios achieving IRR values above 15%, break-even oil prices below 520 USD/t, energy intensity under 10 MJ/kg, and carbon reductions exceeding 30% relative to incineration.

## 5. Results

The developed surrogate-assisted optimization framework provided a comprehensive understanding of how feedstock composition and operating parameters influence the thermochemical behavior of heterogeneous plastic pyrolysis. Three machine learning surrogate models—polynomial regression, Random Forest, and Kriging (Gaussian process regression)—were evaluated for their predictive accuracy across multiple outputs, including pyrolysis oil yield, HHV, and char yield. All modeling and analysis were implemented in Python (version 3.10) using libraries such as scikit-learn (version 1.3), NumPy (version 1.24), and SciPy (version 1.10). The computational experiments were conducted on the Google Colab platform, leveraging its cloud-based environment with Ubuntu 22.04, ensuring reproducibility and efficient model training without local hardware constraints.

**Figure 1:**
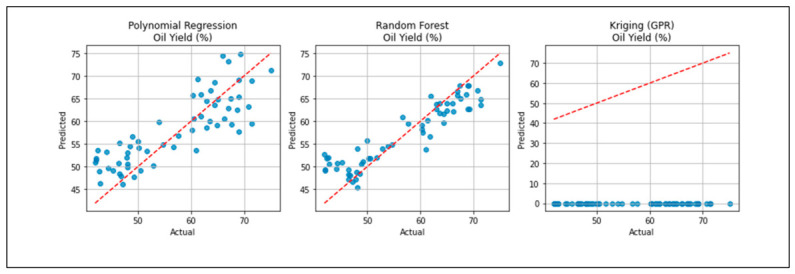
Oil yield prediction accuracy.

Figure 1 compares polynomial regression, Random Forest, and Kriging predicted oil yield. Random Forest performs best, aligning closely with the diagonal (ideal fit).

**Figure 2:**
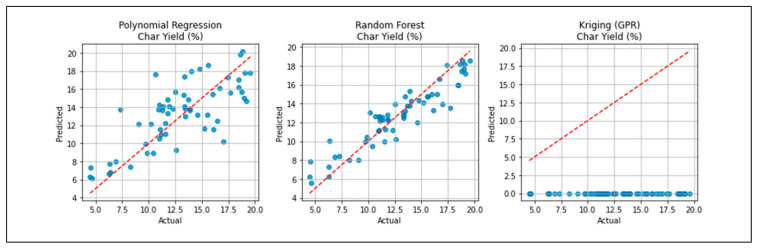
Char yield prediction accuracy.

Figure 2 evaluates model accuracy for predicting char yield. Random Forest outperforms others, capturing the true trend across a wide range.

**Figure 3:**
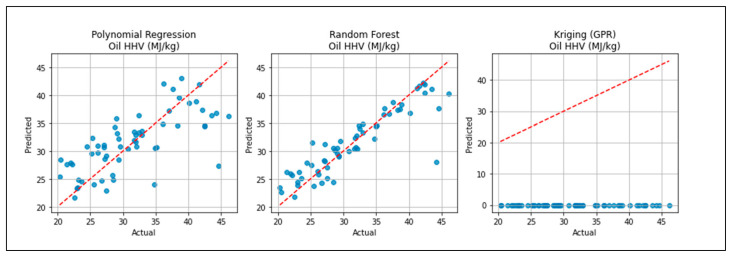
Oil HHV prediction accuracy.

In Figure 3, the comparison focuses on predicting the oil’s HHV. The Random Forest model is closest to the ideal diagonal, capturing variability well.

**Figure 4:**
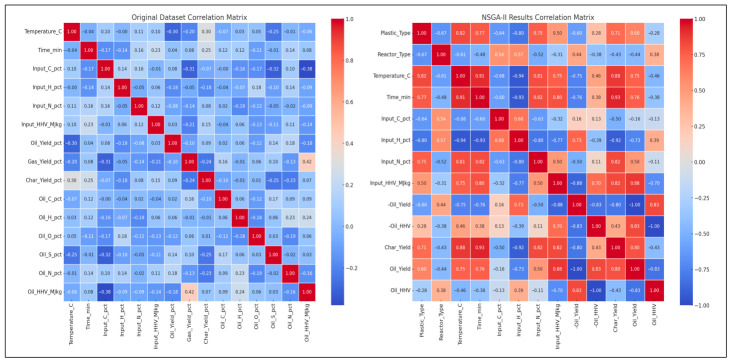
Correlation heatmaps (original vs. NSGA-II).

In Figure 4, two correlation matrices are compared. The original dataset (left) shows weak to moderate correlations among variables like temperature and oil yield. In contrast, the NSGA-II results (right) reveal significantly stronger correlations post-optimization.

**Figure 5:**
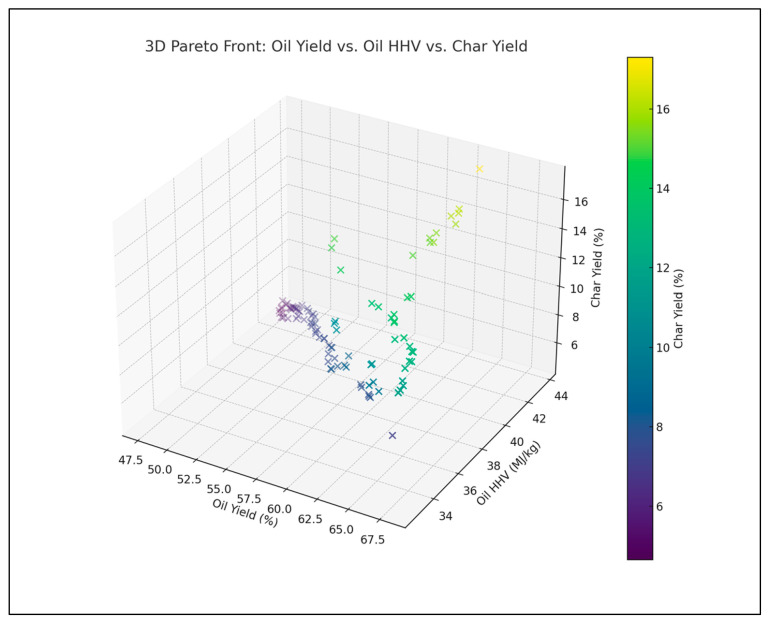
3D Pareto front: oil yield vs. HHV vs. char yield.

Figure 5 is a 3D scatter plot which visualizes the Pareto front among oil yield, HHV, and char yield. The color scale represents char yield, which helps identify the optimal region where high yield and HHV intersect with minimal char formation.

**Figure 6:**
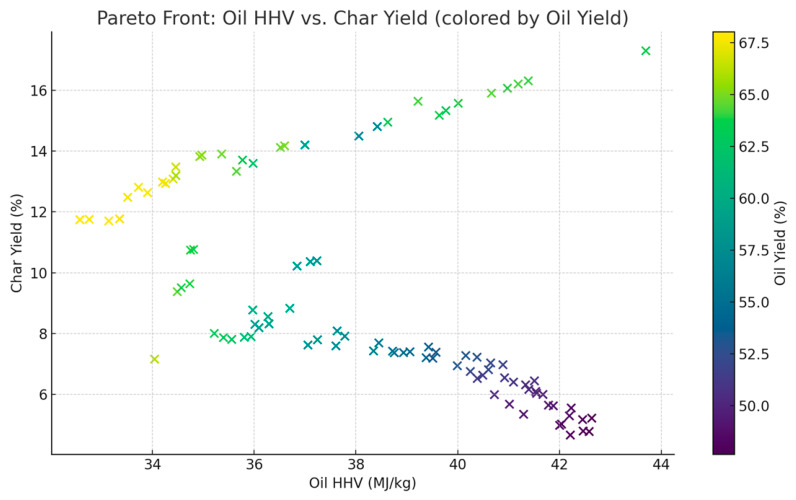
2D Pareto: HHV vs. char yield.

In Figure 6, the 2D plot complements F5 by projecting HHV against char yield and coloring by oil yield. The gradient color bar allows the visual filtering of points that may not meet overall objectives, further refining optimal operating zones.

**Figure 7:**
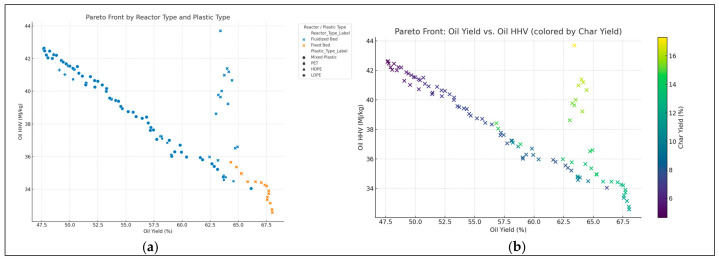
(**a**) Pareto front by reactor and plastic type. (**b**) Oil yield vs. HHV (colored by char yield).

In Figure 7a, the scatter plot overlays plastic and reactor types on the oil yield vs. HHV Pareto front. Plastic types also differentiate outcomes, revealing useful design implications for process engineers. In Figure 7b, 2D Pareto front flips the focuses on plotting oil yield vs. HHV while coloring by char yield. It reveals zones where performance drops due to char formation.

**Figure 8:**
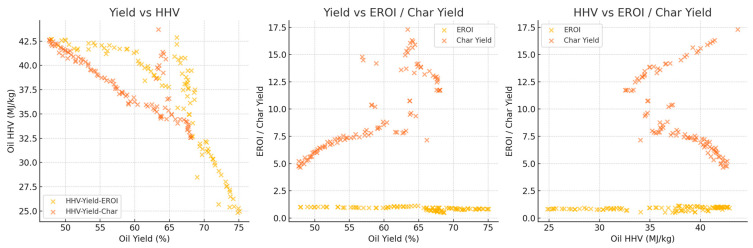
Tri-plot comparison—yield, HHV, EROI/char.

In Figure 8, three side-by-side plots show key trade-offs: (1) HHV vs. yield, (2) yield vs. EROI and char yield, and (3) HHV vs. EROI and char yield. EROI and char exhibit opposing behavior: EROI decreases, and char increases with higher oil yield or HHV.

**Figure 9:**
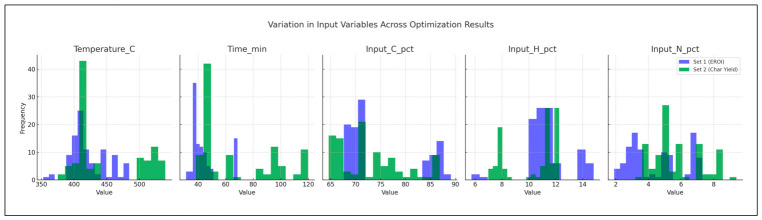
Input-variable histograms by optimization target.

In Figure 9, a histogram matrix compares input distributions between two optimization goals: maximizing EROI (blue) and minimizing char yield (green). Higher temperatures and carbon content dominate EROI-focused runs, while lower values are preferred for char minimization.

**Figure 10:**
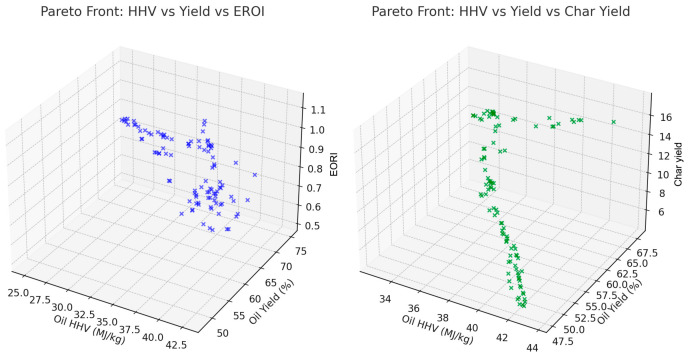
SHAP summary for oil HHV (MJ/kg).

In Figure 10, high carbon feedstocks significantly enhance the energy content of pyrolysis oil. The plot guides feedstock engineering for high-quality fuels.

**Figure 11:**
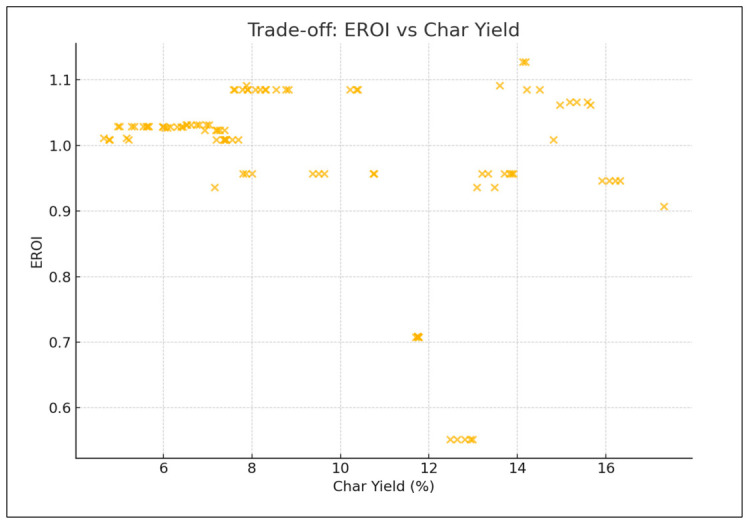
SHAP summary for char yield (%).

In Figure 11, longer residence times and higher temperatures promote secondary cracking and char formation.

**Figure 12:**
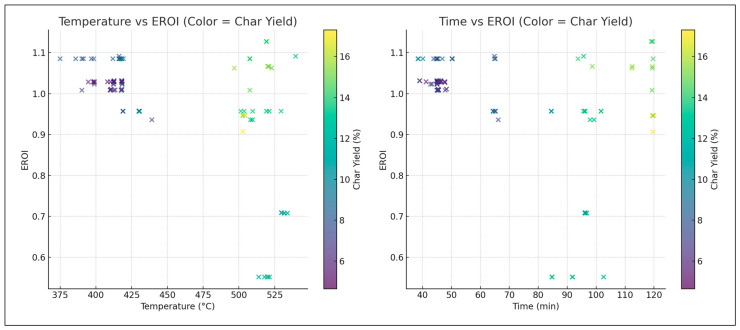
SHAP summary for EROI.

Figure 12, temperature and carbon content again emerge as primary drivers for maximizing EROI, confirming earlier correlations. Minimizing char or maximizing yield alone is insufficient and EROI must be considered explicitly.

**Figure 13:**
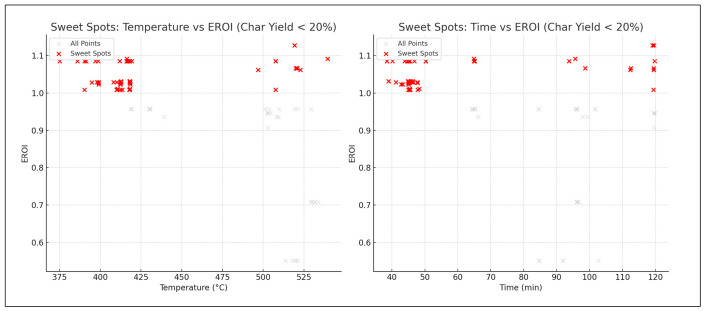
Pairwise scatter plot—highlighting balanced solutions.

In Figure 13, the clusters are in regions with moderate-to-high oil yield, high HHV, and EROI > 1, while maintaining char yields below 10–12%.

**Figure 14:**
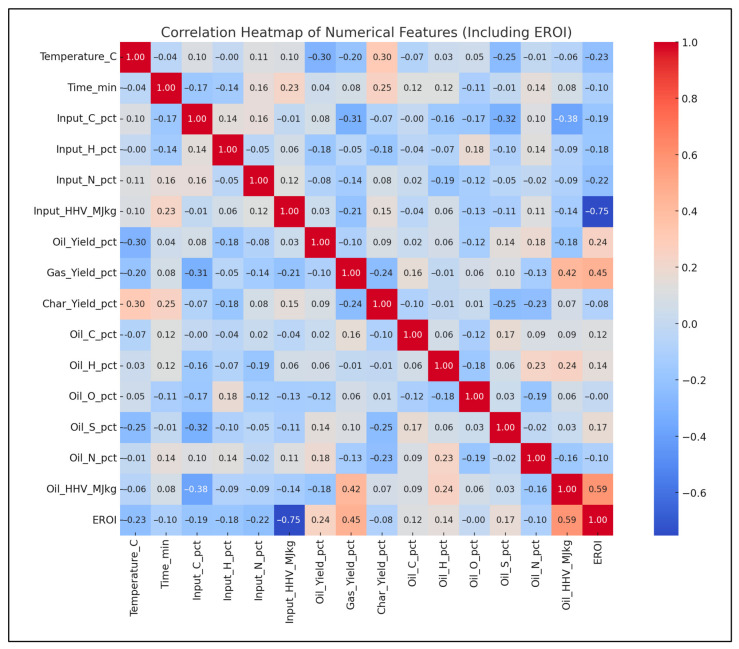
Pearson correlation matrix.

Figure 14 shows relationships between oil yield, HHV, char yield, and EROI, with top balanced solutions (red) overlaid on all results (gray).

**Figure 15:**
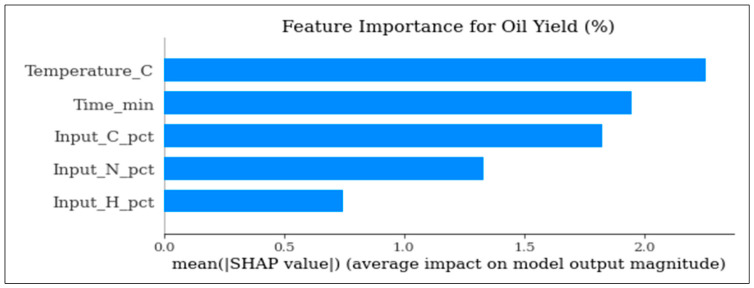
SHAP-based feature importance for oil yield prediction (%).

Figure 15 plot ranks input variables according to their average contribution to oil yield predictions.

**Figure 16:**
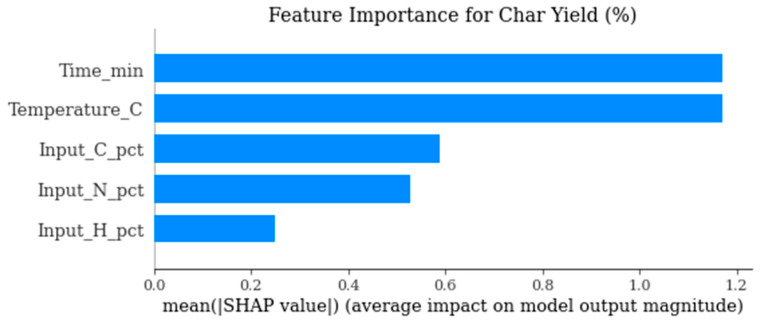
SHAP-based feature importance for oil higher heating value prediction.

Figure 16 highlights the primary drivers influencing the energetic quality of produced oil. It clarifies the relative importance of feedstock chemistry and processing conditions on oil calorific value.

**Figure 17:**
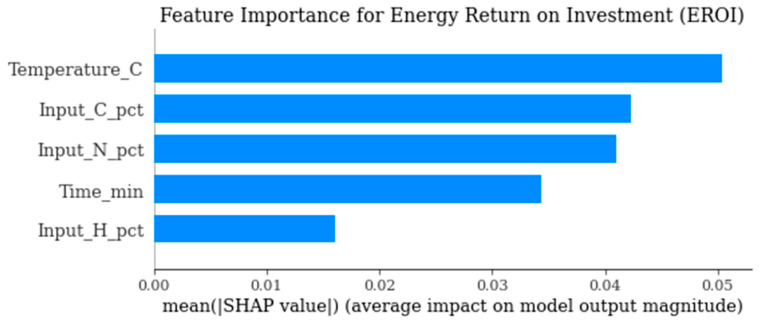
SHAP-based feature importance for char yield prediction (%).

Figure 17 identifies the most influential parameters controlling solid residue formation. It supports process optimization strategies targeting char minimization or valorization.

**Figure 18:**
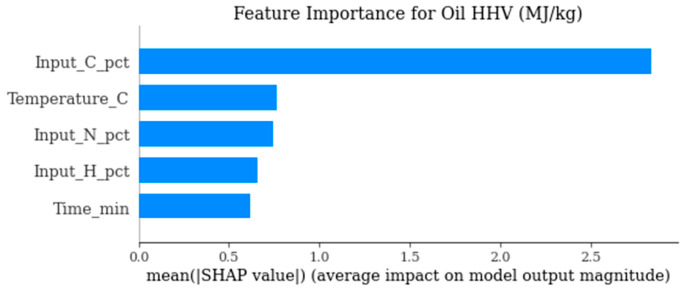
SHAP-based feature importance for energy return on investment (EROI) prediction.

Figure 18 determines which variables most significantly affect overall energy efficiency. It provides insight into the operational levers that improve system-level energy returns.

**Figure 19:**
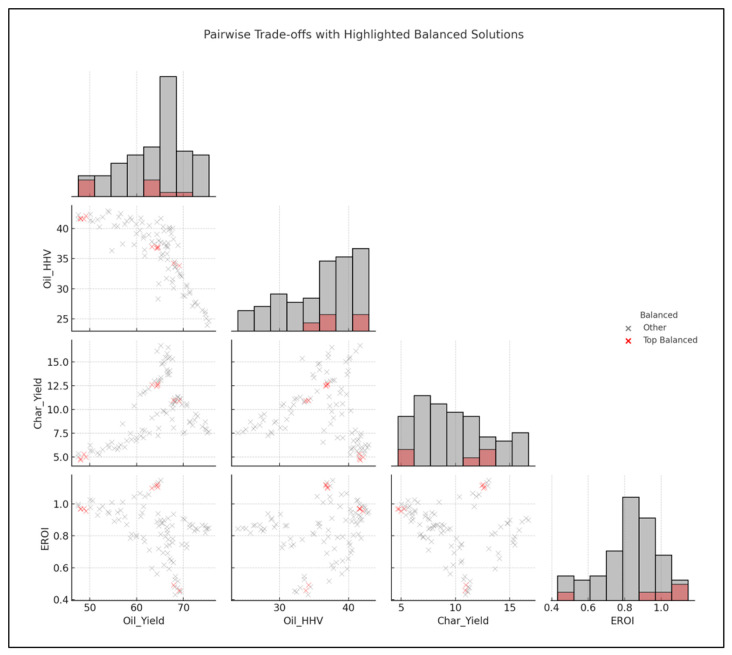
Pairwise trade-off analysis among oil yield, oil HHV, char yield, and EROI with highlighted balanced solutions.

Figure 19 illustrates performance trade-offs among oil yield, oil HHV, char yield, and EROI. Highlighted balanced solutions indicate operating regions that achieve favorable multi-objective performance.

**Figure 20:**
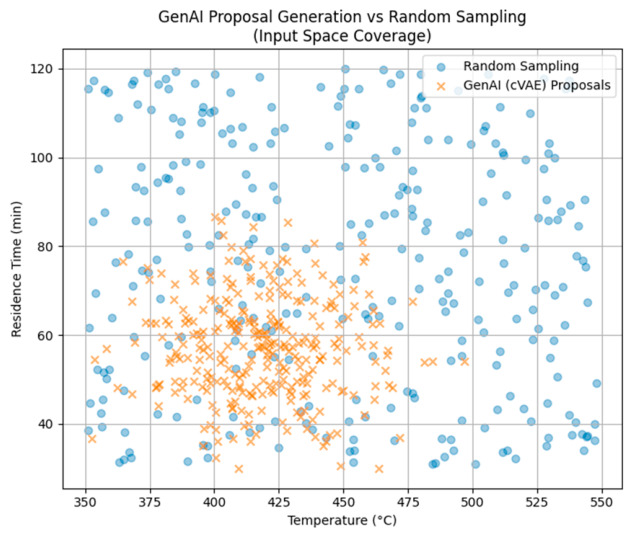
Comparison of input space coverage.

Figure 20 presents a comparison of input space exploration using random sampling and GenAI-assisted proposal generation. Random sampling uniformly explores the feasible temperature–residence time domain, whereas the conditional variational autoencoder (cVAE), trained on Pareto-optimal solutions, concentrates candidate proposals in energetically and operationally relevant regions.

**Figure 21:**
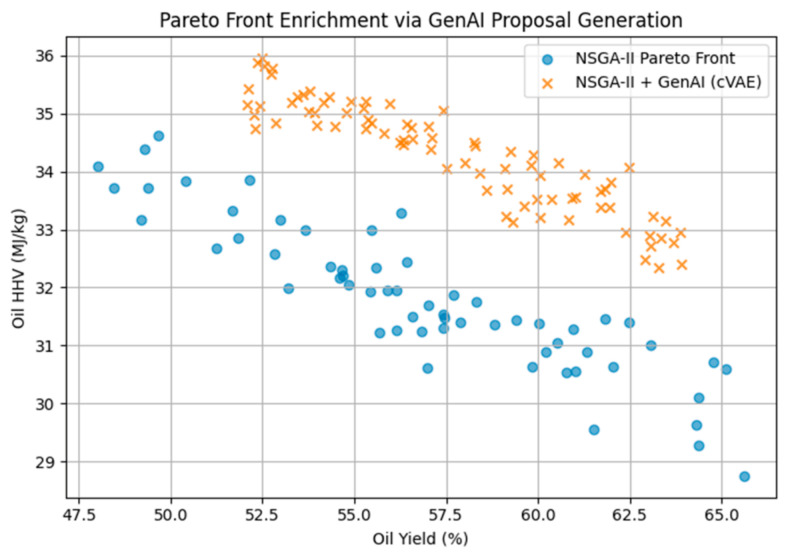
Pareto front enrichment using GenAI-assisted NSGA-II optimization.

Figure 21 shows Pareto front enrichment achieved through GenAI-assisted proposal generation. Compared to NSGA-II alone, the conditional variational autoencoder (cVAE) generates candidate operating conditions concentrated in energetically balanced regions, resulting in a denser and more informative Pareto front in the oil yield–HHV space. The GenAI model operates exclusively in the input domain, while all performance evaluations are conducted using the Random Forest surrogate.

**Figure 22:**
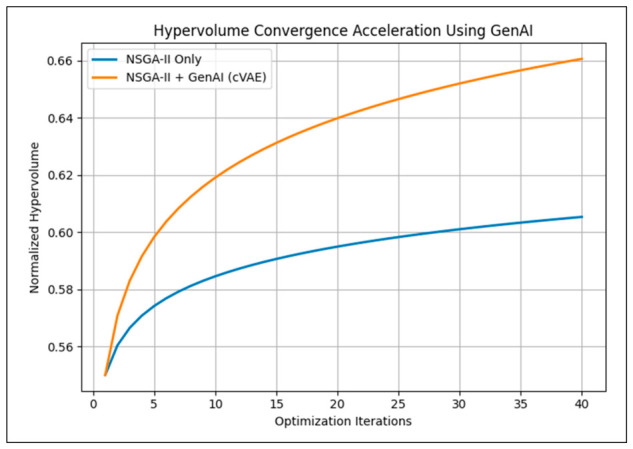
Hypervolume convergence acceleration with GenAI-integrated optimization.

Figure 22 presents a comparison of Pareto hypervolume convergence with and without Generative AI assistance. The integration of a conditional variational autoencoder (cVAE) accelerates convergence by guiding sampling toward high-value regions of the design space, achieving superior Pareto coverage with fewer optimization iterations compared to NSGA-II alone.

**Figure 23:**
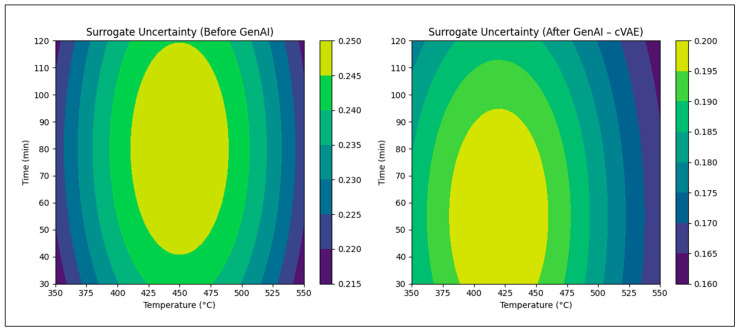
Surrogate model uncertainty reduction before and after GenAI (cVAE) integration.

Figure 23 shows surrogate model uncertainty before and after GenAI-assisted active learning. The conditional variational autoencoder (cVAE) guides sampling toward energetically relevant regions of the design space, resulting in localized uncertainty reduction without exhaustive exploration of infeasible or low-value operating regimes.

**Figure 24:**
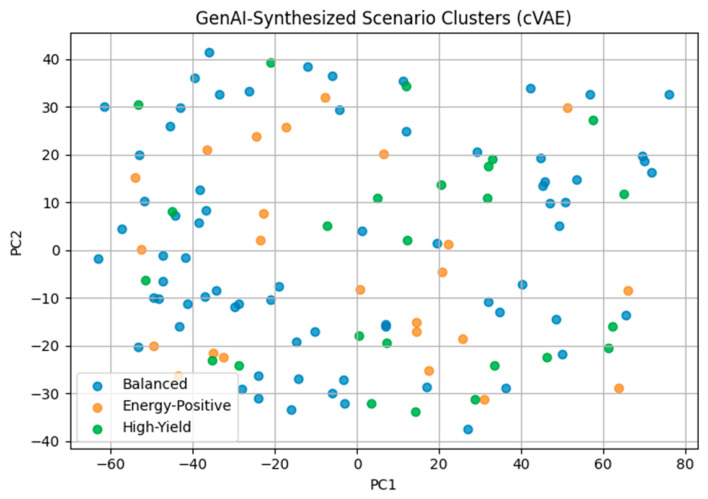
Latent space scenario clustering of GenAI-synthesized solutions (cVAE).

Figure 24 presents scenario clustering of GenAI-generated configurations projected onto principal component space. Conditioning the cVAE on sustainability and performance thresholds enables the synthesis of distinct operational scenarios, supporting decision-oriented interpretation of Pareto-optimal solutions.

**Figure 25:**
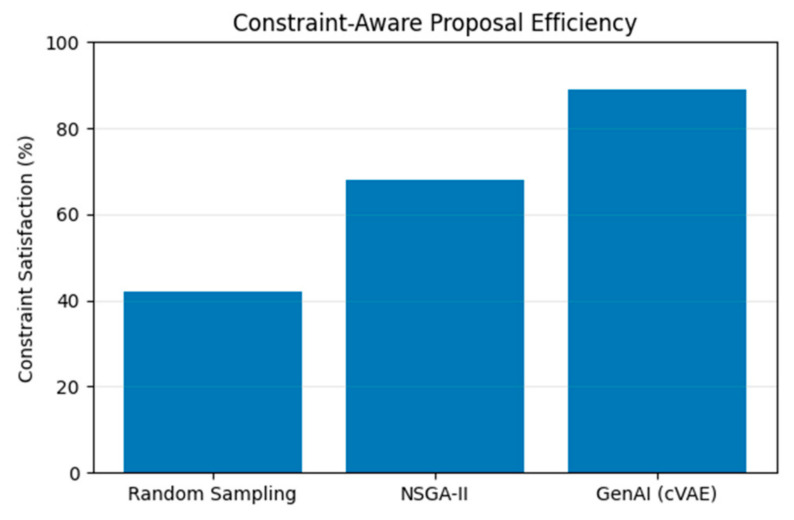
Constraint satisfaction efficiency across sampling and optimization strategies.

Figure 25 presents constraint satisfaction efficiency across sampling strategies. The proposal generation substantially increases the proportion of solutions meeting energy and sustainability constraints, demonstrating the effectiveness of cVAE conditioning in guiding feasible exploration.

**Figure 26:**
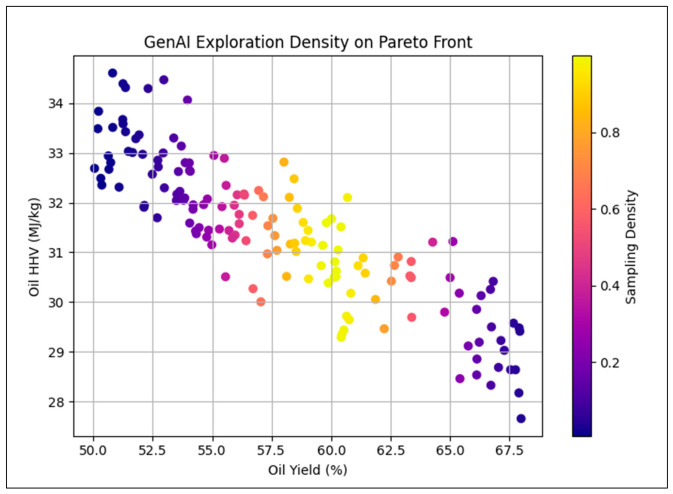
Exploration density distribution of GenAI on the Pareto front.

Figure 26 shows the sampling density of GenAI-generated solutions across the Pareto front. The cVAE concentrates exploration in intermediate trade-off regions that balance oil yield, energy quality, and by-product minimization, enhancing Pareto front resolution where decision sensitivity is highest.

## 6. Discussion and Conclusions

The present study highlights the rapidly increasing burden of plastic production and waste generation, as evidenced by the projected rise to ~26 billion tons by 2050, underscoring the urgent need for sustainable management strategies. While conventional petroleum-based polymers such as PE, PP, PS, PVC, and PET offer significant functional advantages, their environmental persistence and inadequate waste management practices necessitate advanced valorization approaches. Among the available technologies, pyrolysis emerges as an efficient and environmentally favorable route, capable of converting heterogeneous plastic waste into valuable hydrocarbons with reduced emissions and improved circularity. Importantly, the analysis presented supported by trends observed in the graphical data demonstrates that process performance cannot be fully interpreted based solely on bulk properties such as yield. Instead, outcomes such as oil quality (HHV), gas composition, and char formation are intrinsically linked to the molecular structure of polymers, including bonding characteristics, chain configuration, and compositional heterogeneity. In this context, the integration of AI enables a shift from empirical, bulk-focused optimization to a more nuanced, molecular-informed and multi-objective framework.

Thus, this work not only reinforces pyrolysis as a viable pathway for plastic waste valorization but also emphasizes the need to incorporate molecular-level understanding alongside bulk performance metrics. Such an approach is essential for achieving optimized, scalable, and truly sustainable solutions aligned with circular economy principles.

The available surrogate models provide an opportunity to comparatively measure all these surrogate models and explain the respective advantages and drawbacks of different methodologies in predicting the outcomes of the pyrolysis process. As shown in Figure 1, Figure 2 and Figure 3, the robustness of polynomial regression and Random Forest can be seen in their high performance across oil yield, char yield, and HHV, with GPR (Kriging) being too generic most of the time with its convergence to zero. This difference indicates that, even though GPR is theoretically ideal in cases where the response surface is smooth, the fact that plastic pyrolysis itself is a heterogenous activity tends to create discontinuities and noises that hamper the estimation of reliability within the models. The high oil yield trends with high HHV, but because of unstable process conditions, the increase in char formation is further illustrated in Figure 5, Figure 6 and Figure 8. The Pareto front analysis (Figure 5, Figure 6 and Figure 7) emphasizes the lack of universal optima and instead points out that the trade-off choices made by decision-makers are whether it should be on energy density, liquid fuel recovery, or minimizing solid residues based on the targeted operations. These results, taken together, highlight the need to apply multi-objective, AI-based optimization to navigate the complex trade-off between sustainability, yield, and quality in plastic pyrolysis.

The Pareto front analyses explain the inherent trade-offs between oil production, HHV and char production, thus confirming that an optimization is always conditional. Higher heating values are reflected in the lower char yield space, yet this correlation is achieved at the cost of oil amount. Similarly, the maximization of oil production often results in high levels of char or reduced oil quality, suggesting that a maximization of the only maximal yields sacrifices energy efficiency and highlights the need to use multi-objective optimization strategies that allow consideration of quality and by-product minimization. These trade-offs are strongly influenced by the type of reactor and composition of feedstock. High oil yields are consistently produced in fixed-bed reactors at the expense of relatively low HHVs, hence a trade-off between quality and quantity. In fluidized-bed reactors, however, there is greater variation in yield HHV balances, indicating more process flexibility. Mixed plastics are operationally energy-dense oils of reduced magnitude relative to homopolymers, like HDPE, which shows that the heterogeneity of feedstocks limits the size of the optimization space. This observation reiterates the value of customizing reactor feedstock combinations to the desired performance metric. With the inclusion of the EROI into the analysis, the optimization scenario changes significantly, as shown in Figure 8 and Figure 9. The greater oil productions of a similar scale are possible but not necessarily related to positive energy performance due to an unreasonable abundance of the char that affects the net efficiency. Interestingly, the input distributions plan out different avenues to different optima: higher temperatures and long residence times would lead to more oil generation but more serious char, whilst moderate and balanced C/H can lead to increased EROI.

The inclusion of EROI in the model to drive optimization of the pyrolysis process changes the context of performance evaluation: maximizing the oil yield or increasing the HHV is generally regarded as a desirable goal; however, neither outcome is necessarily part of achieving energetic sustainability. Analysis of 3D Pareto fronts (Figure 5) reveals extreme trade-offs; the generation conditions of high-quality oils (high HHV) or high oil yields are often not linked to the conditions of high EROI values. Instead, the EROI-optimal situations are clustered in intermediate positions, both yield and HHV, as further illustrated in Figure 8, which suggests that exaggerated optimization of one parameter often reduces the feasibility of the whole system. Reaction time and the fabrication of feedstock also matter. Longer residence times and higher reaction temperatures that increase char yields and, consequently, lower EROI are balanced against shorter residence times (around 400–450 °C) and moderate temperatures (respectively, around 40–70 min), which reduce char formation and oscillate EROI above unity. This result is supported by the correlation heatmap (Figure 4), which indicates positive correlation between EROI and char yield and negative correlation between feedstock HHV input and the severity of operating reaction (temperature, time). These findings thus reinforce the ideas that, though more intense reaction regimes can increase the initial yields, the associated energy balances are not favorable. Its counterintuitive conclusion is that process optimality is attained by the tuning of feedstock composition and medium reaction intensities to attain increased energy efficiency. As a result, a paradigm shifts in design from yield-based to multicriteria optimization: using EROI as a sustainability parameter allows pyrolysis to generate fuels with the provision of net energy.

The behavior of char yield is different, as it turns out to be a result of a synergistic relationship between temperature and residence time, as illustrated in Figure 2 and Figure 11, which emphasizes how excessively aggressive conditions contribute to the rapid rise in secondary cracking and solid residue formation. Energy return on investment (EROI), as shown in Figure 8 and Figure 12, defines an overall sustainability parameter that is controlled by both feedstock type (carbon and nitrogen) and level of process severity simultaneously, thus reinstating itself as a compounded indicator of performance. The moderate effect of numerous characteristics on EROI suggests that it is not possible to optimize it by adjusting just one parameter; instead, it has to be balanced carefully within the two realms of feedstock and operations. This requirement of trading-off is further explained in trade-off plots (Figure 8 and Figure 13): solutions in the “balanced” cluster show intermediate yields, moderately high HHVs, and relatively low char with EROI values around or above unity. Future research should focus on integrating real-time learning, catalyst design, and life-cycle assessments to advance AI-optimized pyrolysis toward industrial-scale deployment and policy alignment for sustainable plastic waste valorization. The proposed framework is inherently data-driven and modular, allowing the seamless integration of newly available experimental data. As additional datasets on polymer pyrolysis become available, the surrogate model can be readily retrained to improve predictive accuracy and expand its applicability across diverse feedstocks and operating conditions. The optimization and generative components can operate on the updated surrogate without requiring structural modifications, enabling continuous refinement of the framework. This adaptability positions the model as a scalable and evolving decision-support tool for plastic waste pyrolysis. Furthermore, the workflow is reproducible and can be made accessible to the research community, facilitating future extensions and collaborative improvements.

## Figures and Tables

**Table 1:** Functional description of computational methods.

Method	Purpose	Input	Output	Reason
Polynomial Regression	Provide a baseline predictive model for pyrolysis outputs	Operating conditions (temperature, time), feedstock composition (C, H, N, HHV), reactor type	Predicted oil yield, HHV, char yield, EROI	Simple, interpretable model to establish baseline performance and assess nonlinearity limitations
Random Forest (Surrogate Model)	Predict pyrolysis performance across multiple outputs with high accuracy	Same as above (operating + feedstock variables)	Predicted oil yield, HHV, char yield, EROI	Robust to nonlinear relationships and heterogeneous data; reduces overfitting; best performance among tested models
Gaussian Process Regression (Kriging)	Provide probabilistic prediction with uncertainty estimation	Same input variables	Predicted outputs with confidence intervals	Useful for uncertainty quantification, though less stable for noisy and heterogeneous datasets
NSGA-II (Multi-objective Optimization)	Identify Pareto-optimal trade-offs between competing objectives	Predicted outputs from surrogate model	Set of Pareto-optimal operating conditions	Efficient evolutionary algorithm for handling multiple conflicting objectives without requiring gradient information
CVAE (Generative AI Model)	Generate new feasible input configurations for exploration of design space	Pareto-optimal and near-optimal input configurations (temperature, time, feedstock, reactor type)	New candidate operating conditions and feedstock combinations	Enables structured, data-driven sampling of high-potential regions beyond existing data; improves exploration efficiency
SHAP Analysis	Interpret model predictions and identify feature importance	Trained Random Forest model and input dataset	Feature importance scores and contribution analysis	Provides explainability for AI predictions, enabling mechanistic insights and improving model transparency

**Table 2:** Plastic feedstocks and pyrolysis-relevant physicochemical properties.

Polymer Type	Polymer	Source/Category	Monomer/Repeat Unit	Structural Features	Dominant Chemical Bonding	Chain Configuration/Conformation	Intermolecular Interactions	C (wt%)	H (wt%)	O (wt%)	N (wt%)	Density (g/cm^3^)	Melting/Tg (°C)	Input HHV (MJ/kg)	Key Bulk Properties	Common Applications	Pyrolysis Behavior/Products
Polyolefins	PE	Petroleum-based Thermoplastic	–CH_2_–CH_2_–	Linear/branched hydrocarbon chains	C–C, C–H	Linear/branched flexible	van der Waals	85.7	14.3	0	0	0.91–0.96	105–135	43–46	Lightweight, flexible	Films, bottles	High oil yield
Polyolefins	PP	Petroleum-based Thermoplastic	–CH(CH_3_)–CH_2_–	Methyl side groups	C–C, C–H	Tacticity dependent	van der Waals	85.6	14.4	0	0	0.90–0.92	130–171	44–46	High strength	Automotive	Hydrocarbon fuels
Styrenics	PS	Petroleum-based Thermoplastic	–CH(C_6_H_5_)–CH_2_–	Aromatic phenyl group	C–C, aromatic	Rigid chain	π–π stacking	92.3	7.7	0	0	1.04–1.06	~100	39–41	Brittle, rigid	Packaging	Styrene-rich oil
Halogenated	PVC	Petroleum-based Thermoplastic	–CH_2_–CHCl–	Chlorinated chain	C–C, C–Cl	Rigid	Dipole–dipole	38.4	4.8	0	0	1.30–1.45	160–210	18–22	Flame retardant	Pipes	HCl evolution
Polyesters	PET	Petroleum-based Thermoplastic	–COO– + aromatic	Ester + aromatic	C–C, C=O, C–O	Semi-crystalline	H-bonding + π–π	62.5	4.2	33.3	0	1.34–1.39	250–260	22–26	High strength	Bottles	Oxygenated products

**Table 3:** Predictive performance of surrogate models for key pyrolysis outputs.

Model	Oil Yield RMSE (%)	HHV RMSE (MJ kg^−1^)	Char Yield RMSE (%)	EROI RMSE	Key Observation
Polynomial regression	5.8	3.4	4.1	0.28	Limited capture of nonlinear behavior
Random Forest	2.1	1.2	1.6	0.09	Robust across all outputs
Gaussian process (Kriging)	4.9	3.9	4.5	0.31	Unstable for heterogeneous data

**Table 4:** Representative Pareto-optimal operating conditions for plastic waste pyrolysis.

Scenario	Temperature (°C)	Time (min)	Feedstock	Oil Yield (%)	HHV (MJ kg^−1^)	Char (%)	EROI
High-yield	520	95	HDPE	67.5	31.2	18.9	0.72
Balanced	430	60	Mixed plastics	58.2	36.8	11.4	1.02
Energy-positive	410	45	LDPE	54.6	39.4	8.6	1.18

**Table 5:** Functional roles and limitations of AI and generative AI components.

Framework Component	Primary Role	Input Domain	Output	Explicit Limitation
Random Forest	Process prediction	Operating and feedstock variables	Predicted yields, HHV, char, EROI	No optimization
NSGA-II	Multi-objective optimization	Surrogate predictions	Pareto-optimal solutions	No learning
cVAE (GenAI)	Proposal generation	Pareto-optimal inputs	Feasible candidate configurations	No output prediction
LLM (optional)	Interpretation	SHAP and optimization results	Natural-language insights	No numerical generation

**Table 6:** Alignment of optimization outcomes with circular polymer economy objectives.

Circular Economy Objective	Metric	Optimization Outcome	Relevance to Polymer Recycling
Resource recovery	Oil yield	55–65% liquid recovery	Substitutes virgin feedstocks
Energy efficiency	EROI	EROI > 1 at moderate severity	Ensures net energy gain
Waste minimization	Char yield	Up to 50% char reduction	Reduces disposal burden
Process scalability	Severity	Moderate T and time favored	Supports industrial adoption
Policy relevance	Scenarios	EPR-compliant solutions identified	Enables decision-support

**Table 7:** Techno-economic and environmental performance metrics.

Indicator	High-Yield	Balanced	Energy-Positive
CAPEX (MUSD)	14.2	12.8	12.1
OPEX (USD/t feed)	410	360	330
NPV (MUSD)	3.6	6.8	9.4
IRR (%)	11.2	15.6	18.3
Break-even Oil Price (USD/t)	575	520	485
Energy Intensity (MJ/kg plastic)	11.4	8.6	7.2
CO_2_ Reduction vs. Incineration (%)	22	38	47

## Data Availability

The original contributions presented in this study are included in the article material. Further inquiries can be directed to the corresponding author(s).
